# Implementation of evidence-based practice and associated factors among nurses and midwives working in Amhara Region government hospitals: a cross-sectional study

**DOI:** 10.1186/s12978-021-01096-w

**Published:** 2021-02-12

**Authors:** Asrat Hailu Dagne, Mekonnen Haile Beshah, Bekalu Getnet Kassa, Eyaya Habtie Dagnaw

**Affiliations:** Department of Midwifery, Debre Tabor University, Debre Tabor, Amhara Region Ethiopia

**Keywords:** Implementation, Evidence-based practice, Nurses/midwives work index

## Abstract

**Background:**

Implementation of evidence-based practice is crucial to enhance quality health care, professional development, and cost-effective health service. However, many factors influence the implementation of evidence-based practice. Therefore, this study aimed to assess the implementation of evidence-based practice and associated factors among nurses and midwives.

**Methods:**

Institutional-based cross-sectional study design was conducted to assess the implementation of evidence-based practice and associated factors from February 15 to March 15, 2019, among 790 nurses and midwives. Data were entered into EpiData version 3.1 then exported to SPSS version 20 for statistical analysis. Categorical variables were presented as frequency tables. Continuous variables were presented as descriptive measures, expressed as mean and standard deviation. Cronbach’s alpha was used to measure reliability, mean, standard deviation, and inter-items correlation of the factors. Independent variables with a probability value (P-value) of less than 0.2 in the Chi-square analysis were entered in the multivariable logistic regression model. Statistically significant associated factors were identified at probability value (P-value) less than 0.05 and adjusted odds ratio with a 95% confidence interval.

**Results:**

The mean age of participants was 28.35 (SD ± 4.5) years. This study revealed that 34.7% (95% CI 31.5–38%) of participants implemented evidence-based practice moderately or desirably. Age of participants (AOR = 5.98, CI 1.34–26.7), barriers of implementation of evidence-based practice (AOR = 4.8, CI 2.2–10.6), the attitude of participants (AOR = 5.02, CI 1.2–21.5), nursing/midwifery work index (AOR = 3.9, CI 1.4–10.87), self-efficacy of implementation of evidence-based practice skills (AOR = 12.5, CI 5.7–27.5) and knowledge of participants (AOR = 3.06, CI 1.6–5.77) were statistically significant associated factors of implementation of evidence-based practice

**Concussion:**

Implementation of evidence-based practice of nurses and midwives was poor. Age of participants, barriers of implementation of evidence-based practice, the attitude of participants, self-efficacy of implementation of evidence-based practice skills, nursing/midwifery work index, and knowledge of participants were found to be predictors of implementation of evidence-based practice. Insufficient time and difficulty in judging the quality of research papers and reports were the most common barriers to the implementation of evidence-based practice.

## Background

Implementation of evidence-based practice (IEBP) is defined as the use of the best available, current, valid, and relevant evidence like research, work experience, and updated standard guidelines in the clinical decision-making practice [1–3]. Evidence-based practice (EBP) is implemented when it relies on interpreting currently available findings and applying rational scientific research [4]. IEBP is crucial to increase the best patient outcomes [5] and it reduces patient pain, hospital stay, and ulcers due to pressure [6].

It is about 26 years since EBP has been introduced by a medical working group that published the first paper on evidence-based practice in 1992 [7]. Worldwide, the quality of research and standard guidelines engaging in evidence-based behavior is low. In addition to this, most factors influencing IEBP are not well identified and there is a call for further research to be done globally [8]. Multifaceted interventions like training and educational meetings on research utilisation have improved evidence-informed clinical decision-making practice and patient outcomes [8]. Nowadays, more developments are advocated and international organizations are encouraging the need for IEBP [9]. However, nurses and midwives have been dependent on experts’ opinions of seasoned nurses and midwives without using currently available evidence [10]. The traditional ways of EBP may not only outdated but also unsecured [5]. Traditional-based knowledge can be associated with favored thinking that leads to mistakes [11].

Careful managing resources and the demand for maximum quality healthcare increases the pressure on health care professionals to ensure evidence-based clinical decision-making practice. Increasing volume of research information, availability of sophisticated medical care, client expectation to get the best possible care, and the rising health care expenditure compel the governments around the globe to embrace EBP. Therefore, EBP is an important way to deliver quality healthcare [10]. EBP is well known in the United Kingdom, Canada, and the United States of America. The outcome measurement and effectiveness of IEBP in public service are seen by the government and citizens in the settings of high-income countries [11]. There is a view that evidence-based approaches can have a strong effect on outcomes in low-income countries. If a call to action to create an EBP for health professionals in these countries is provided, better IEBP in the health care system of these countries could dramatically improve healthcare delivery through the intervention on barriers of IEBP [11, 12].

Poor access to good quality research and lack of time are the most frequently reported barriers. In addition to this, there is a lack of collaboration between researchers and policymakers, and improved relationships and skills [13, 14]. Shortage of resources, poor health care system and limited intervention are challenges of IEBP in the health care of low and middle-income countries [10]. Utilisation of research findings is limited to educational or academic institutions in Africa and there is a lack of knowledge to improve quality health care delivery, the standard of care, and quality of life through IEBP [15]. The use of experienced knowledge and currently available updated standard guidelines are recommended for organizations to ensure evidence-based quality health care. However, the implementation of this evidence is not known. Factors affecting IEBP are different according to the study settings [16].

There is a challenge for policymakers to use evidence for developing policy and strengthening the healthcare system [17]. There remains a dearth of research examining IEBP and studies are less reliable in this country. The findings of this study could help program managers, stakeholders, and health service providers to improve IEBP and quality healthcare service through intervention. It could also contribute to getting new research ideas for researchers. Therefore, this study was aimed to determine the implementation of evidence-based practice and identify independent predictor variables of IEBP among nurses and midwives.

## Methods

### Study design and setting

An institutional-based cross-sectional study design was conducted to assess the implementation of evidence-based practice and associated factors from February 15 to March 15, 2019, among nurses and midwives working in Amhara Regional State public hospitals. The study considered three specialized hospitals, four general hospitals, and 20 primary hospitals. Each of the specialized hospitals serves more than three million people. Each of the general and primary hospitals serves two million and one hundred fifty thousand people respectively.

### Sample size determination and sampling technique

Nurses and midwives working in Amhara Regional State public hospitals were the study population. The sample size was calculated using single population proportion formula and the required sample size for this study was determined using the following assumptions; desired precision (d) = 5%, Confidence level = 95% (Ζα/2 = 1.96 value), and 57.6% of nurses implemented evidence-based practice in their clinical practice in Tikur Anbessa Specialized Hospital [18]. Therefore, the calculated total sample size from the largest outcome variable is 375.28. Considering the design effect of 2, the sample size was 751. Adding a 10% non-response rate, the final sample size was n = 826.

The regional state has 13 zones. From these administrative zones, thirty-one percent (four zones) were selected randomly by lottery method. There were 27 public hospitals in the selected zones and 27 public hospitals were included in the study. In the selected hospitals, there were 1567 eligible nurses and midwives. The sampling frame, which includes a list of 1567 nurses and midwives, was prepared using a registration file (staff folder). Finally, participants from each hospital were selected using a computer-generated random number sampling technique from the sampling frame.

### Participants

Nurses and midwives working in private health institutions were excluded from the study to avoid double counting. This is because public health workers could be part-timers in private institutions. Moreover, nurses and midwives whose level of education was diploma were not familiar with research due to a lack of research method and epidemiology in their curriculum. Participants who were ill and unable to respond during the study period were also excluded from the study. A sample of 790 nurses and midwives whose educational status was degree and master were included to participate in this study during the study period.

### Measurements

All questionnaires were developed following a detailed literature examination [18–23]. Valid and reliable items were considered from different literature. Questionnaires had sub-section of IEBP, socio-demographic, organization, attitude, self-efficacy of IEBP skill, barriers of implementing EBP, supporting factors for IEBP, and nursing/midwifery work index related characteristics. Twenty professional and language experts reviewed these previously used questions in different studies. Moreover, the questions were prepared first in English then translated to Amharic, and questions were also retranslated back to English for consistency.

Eighteen statements developed by Gerrish [19] and the EBP implementation scale were also developed by Melnyk and Fineout-Overholt [20]. These questionnaires were used to assess the implementation of evidence-based practice. The questionnaires allow the participants to respond to each of the items on a 5 point frequency scale by indicating how often in the past eight weeks participants performed each of the items with the total score ranging from 18 to 90. Questionnaires were also linked to the actual implementation of EBP, such as, how often do you implement evidence-based practice towards each question?”, or “used evidence to change your clinical practice”. The responses for the alternative were 1 = never (1 time before eight weeks), 2 = rarely (1–3 times within the past 8 weeks), 3 = sometimes (4–6 times), 4 = often (7–8 times within the past 8 weeks) and 5 = always (greater than 8 times within the past 8 weeks) [21]. Moreover, some questionnaires developed from the literature were also modified and reviewed to consider updated guidelines, books, hospital protocols, and experts’ opinions that are used as a source of information and knowledge. Thus, the use of these sources of information and knowledge in the clinical and healthcare practice is the element of evidence-based practice.

The attitude of participants towards IEBP, barriers of implementation of EBP, and nursing/midwifery work index related questionnaires were answered as strongly agree, agree, neither agree nor disagree, disagree, and strongly disagree on a five-point Likert scale [22, 23]. Questionnaires related to self-efficacy of IEBP skills were answered as excellent, very good, good, satisfactory, and poor (nine items on a 5-point Likert scale) [22] and supporting factors for IEBP has six items which were answered as most important, important, neither important nor unimportant, least important, and not important on a 5-point Likert scale [22].

The IEBP of participants was measured from the total amount of answers to eighteen IEBP related questionnaires on a 5-point Likert scale, with a minimum score of 18 and the greatest score of 90. The attitude of participants towards IEBP was measured from the total amount of answers to six attitude-related questionnaires on a 5-point Likert scale, with a minimum score of 6 and the greatest score of 30. Self-efficacy of IEBP skills, and barriers of IEBP of participants were measured from the total amount of answers to nine self-efficacy of IEBP skill-related questionnaires and nine barriers of IEBP related questionnaires on a 5-point Likert scale, with a minimum score of 9 and the greatest score of 45. Nursing/midwifery work index of participants was measured from the total amount of answers to 31 nursing/midwifery work index related questionnaires on a 5-point Likert scale, with a minimum score of 31 and the greatest score of 155. The IEBP, the attitude of participants towards IEBP, the self-efficacy of IEBP skills, the barriers of IEBP and the nursing/midwifery work index were considered ‘poor (unfavorable)’, ‘moderate’, and ‘desirable if the participants answered below 60%, 60–80% and greater than 80% for the IEBP, the attitude, the self-efficacy of IEBP skills, the barriers of IEBP, and the nursing, and midwifery work index questions, respectively [22].

### Data collection

Data collectors were selected based on the criteria of previous exposure to data collection and the level of education. Their level of education was a degree, master and Ph.D. They were trained to be familiar with the aim and the methods of the research. To simplify data collection and data handling, data were collected via face-to-face interview technique using semi-structured questionnaires. The pre-test was conducted among 41 (5%) of participants at Woldia Hospital before the actual data collection day. Data collection was started with a greeting and good communication. Data quality was checked at the time of the interview during data collection. The content validity and internal reliability of the questionnaires were assessed using Cronbach’s alpha coefficients. The Cronbach’s alpha coefficient was between 0.698 and 0.98. The supervisors and investigators were closely supervising the performance of data collectors for the actual data collection. Data were checked again after data collection for completeness and internal validity.

### Statistical analysis

The investigators and supervisors checked the data manually for completeness. Data were entered into EpiData version 3.1 then exported to SPSS version 20 for statistical analysis. Categorical variables were presented as frequency tables. Continuous variables were presented as descriptive measures, expressed as mean and standard deviation. Cronbach’s alpha was used to measure reliability, mean, standard deviation, and inter-items correlation of factors. A principal components analysis was employed to identify important variables that explain the total variance of the factors. A chi-square test was done to identify factors associated with the dependent variable. Independent variables with a probability value (P-value) of less than 0.2 in the Chi-square analysis were entered in the multivariable logistic regression model to identify the independent predictors of the implementation of evidenc-based practice. Crude and adjusted odds ratios were used to identify the strength and direction of the association at a 95% confidence interval. A P-value of less than 0.05 was used to decide the significance of the association.

### Operational definitions

#### Implementation of evidence-based practice

This means that respondents implement evidence-based practice when they score greater than 60% (moderately or desirably) [19, 20, 22].

#### No implementation of evidence-based practice

No implementation of evidence-based practice means respondents’ score was less than 60% (poor).

#### Knowledgeable

Half and more than half of the questions were answered by the respondent from the total of knowledge-related questions [18].

#### Not knowledgeable

Half and more than half of the questions were not answered by the respondents from the total of knowledge-related questions.

*The term mild and severe* is used instead of poor and desirable to make sense for the meaning of barriers of IEBP.

## Result

### Socio-demographic characteristics

Out of the expected 826 participants, 790 of them gave a complete response with a response rate of 95.64% and the non-response was due to quit to participation. The mean age of the participants was 28.35 (SD ± 4.5) years. More than half (54.3%) of the study participants were between the age of 25–29 years. About 52% of the study participants were single or not married and 379 (48%) of them were married. About two-thirds (65.4%) of the participants were a nurse and 273 (34.6%) of them were midwives. Most of the participants (97.1%) were BSc nurses or midwives and 23 (2.9%) of participants were MSc nurses or midwives. Regarding the participants’ source of income, 771 (97.6%) of them got their income from salary only, and 19 (2.4%) of them got their income from monthly salary and private business (private clinic and drug vendor). The remaining frequency and percentage of socio-demographic characteristics are presented on the next page (see Table 1).

**Table 1:** Socio-demographic characteristics of nurses and midwives working in Amhara Region public hospitals, North Ethiopia, 2019

Variables	Frequency (n = 790)	Percent (%)
Sex		
Male	416	52.7
Female	374	47.3
Age		
20–24	128	16.2
25–29	429	54.3
30–34	149	18.9
35–54	84	10.6
Position		
Staff nurse	459	58.1
Staff midwife	254	32.2
Head nurse and head midwife	77	9.7
Year of experience		
1–5 years	537	68
6–10 years	175	22.2
11 and above years	78	9.8
Religion		
Orthodox	709	89.7
Others	81	10.3
Ethnicity		
Amhara	743	94.1
Others	47	5.9
Income per month		
100–133 US dollar	153	19.4
134–200	237	30
201–333	227	28.7
> 333	173	21.9

Others (religion) = Muslim, protestant, catholic, and Adventist

### Composite variables related characteristics

About two-thirds of the participants had a total IEBP score below 60% (poor). From the total participants, 33.2% of nurses and midwives had a total IEBP score of 60–80% (moderate) and 1.6% of them had greater than 80% (desirable). From the total sample, 396 (50.1%) of participants were knowledgeable whereas 394 (49.9%) of them were not knowledgeable. Among the total respondents, 374 (47.3%) of them claimed poor nursing/midwifery work index whereas 355 (44.9%) and 61 (7.7%) of them had moderate and desirable nursing/midwifery work index respectively. The remaining composite variables related characteristics are presented on the next pages (see Tables 2, 3, 4, 5).

**Table 2:** Attitude of nurses and midwives towards the implementation of evidence-based practice in Amhara Region public hospitals, North Ethiopia, 2019

Items	Mean	SD
Clinical decision-making practice based on evidence is time-saving	3.05	1.26
Practicing a new clinical approach is preferable to the existing evidence (traditional method) for clinical practice	3.68	1.08
Time allocation in a work schedule for the use of research, guidelines, hospital protocols, textbooks, and experts experience improve implementation of evidence-based practice	3.92	1.02
You accept comments provided by your colleagues that are based on established evidence	3.99	0.9
Research articles from trusted sources are relevant to your daily practice	4.02	0.96
Evidence-based practice is fundamental to professional practice	4.04	1.04
Total	3.78	0.7

**Table 3:** Self-efficacy of IEBP skills of nurses and midwives towards the implementation of evidence-based practice in Amhara Region public hospitals, North Ethiopia, 2019

Items	Mean	SD
You use the checklist to assess how to use research articles, updated guidelines, textbooks, and hospital protocols	2.39	1.06
You identify clinical problems according to currently available evidence	2.62	1.3
You review organizational information and your clinical decision-making practice	2.771	1.19
You use research findings, updated guidelines, hospital protocols, and textbooks to change your clinical decision-making practice	2.772	1.09
Conduct online searches (using databases and web search engines)	2.95	1.18
You have translated a clinical problem into a well-formulated clinical question	3.25	0.995
You analyzed research findings guidelines, hospital protocols, textbooks, and senior experience before you have used the evidence for clinical decision-making practice	3.34	1.06
You have been involved in monitoring and evaluation of clinical practice	3.46	1.05
Apply an intervention based on the most applicable and currently updated evidence like guidelines, hospital protocols, and research findings	3.63	0.98
Total	3.02	0.71

**Table 4:** Barriers of implementation of evidence-based practice of nurses and midwives working in Amhara Region public hospitals, North Ethiopia, 2019

Items	Mean	SD
Difficulty in determining the applicability of research findings	3.5	1.12
Inability to understand statistical terms used in research articles	3.54	1.19
Insufficient time at the workplace to implement changes in their current practice	3.54	1.14
Inadequate understanding of terms used in research articles	3.6	1.18
Inability to properly interpret the results of research studies	3.63	1.18
Inability to implement recommendations of research studies into clinical practice	3.7	1.2
Insufficient resources (e.g. equipment, materials) to implement EBP	3.7	1.17
Difficulty in judging the quality of research papers and reports	3.75	1.2
Difficulty in finding time to search for and read research findings and reports, guidelines, hospital protocols, and books	3.89	1.15
Total	3.66	1.042

**Table 5:** Implementation of evidence-based practice of nurses and midwives working in Amhara Region public hospitals, North Ethiopia, 2019

Items	Mean	SD
How often do you use systematic reviews report in your clinical practice?	1.45	1.013
How often you share research evidence with patients/family members?	2.15	1.14
How frequently you access systematic review databases?	2.37	0.9
How often you critically appraise evidence from a research study?	2.54	1.05
How often you share currently used research evidence, updated guidelines, protocols, and textbooks for your colleague?	2.58	1.05
How often have you changed your practice based on patient outcome data?	2.69	0.94
How often you share currently available evidence with multidisciplinary team members?	2.7	1.1
How often you use trusted current research evidence to change your clinical practice?	2.74	1.12
How often you evaluate clinical practice by collecting patient outcome data?	2.77	1.07
How often you share evidence-based practice guidelines with your colleagues?	2.83	1.03
How often you evaluate the outcomes of a clinical practice change?	2.93	1.034
How frequently you promote the use of the evidence-based practice to your colleagues?	3.09	1.05
How often you perform clinical practice based on midwives’/nurses’ competency?	3.12	1.28
How often do you use the internet to search research articles and guidelines to make a clinical decision?	3.17	1.1
How often you evaluate your clinical practice according to scientific explanation?	3.28	1.13
How often you use world health organization or national guidelines, hospital protocols, and textbooks to make clinical decisions?	3.29	1.1
How often you use local policy and protocols to make clinical decisions?	3.41	1.1
How frequently you read scientific articles, updated guidelines, hospital protocols, and textbooks?	3.7	1.013
Total	2.8	0.5

### Attitude of participants

The internal reliability (Cronbach's Alpha) of attitude-related items was 0.703. The most important attitude towards IEBP was participants believed that evidence-based practice is fundamental to professional practice. However, participants did believe less likely that clinical decision-making practice based on evidence is time-saving (it was the least important factor). The mean attitude score of participants was 75.6% and it was taken as a moderate attitude towards the implementation of EBP (see Table 2). Among the total participants, 76(9.6%), 459 (44.2%), and 255 (32.3%) of them had a poor, moderate, and desirable attitude towards the implementation of evidence-based practice respectively.

### Self-efficacy of IEBP skills

The Cronbach’s alpha of the items was 0.82. Applying an intervention based on the most applicable and currently updated evidence like guidelines, hospital protocols, and research findings was the item with the highest mean score of nurses, and midwives towards Self-efficacy of IEBP skill. The use of a checklist to assess how to use research articles, updated guidelines, textbooks, and hospital protocols was the factor with the least mean score of nurses and midwives towards Self-efficacy of IEBP skills (see Table 3). The mean self-efficacy of IEBP skills of nurses and midwives was 60.4% and it was considered as moderate self-efficacy of implementation of EBP skills. Factor analysis showed that 62.24% total variance of self-efficacy of IEBP skill scale was explained by 2 variables with the Eigen values of 3.88 and 1.72 for the first and second variable respectively. From the total participants, 349 (44.2%) of them had poor Self-efficacy of IEBP skills whereas 389 (49.2%) and 52 (6.6%) of them had moderate and desirable Self-efficacy of IEBP skills respectively.

### Barriers of implementation of evidence-based practice

Cronbach's Alpha of barrier to IEBP scale was 0.969. The most important factor of the barrier of IEBP was difficulty in finding time to search for and read research articles and reports, guidelines, hospital protocols, and standard books. The least important factor was difficulty in determining the applicability of research findings, guidelines, hospital protocol, books, and experts’ experience (see Table 4). The mean barrier of IEBP of participants was 72.3% and it was considered as a moderate barrier to the implementation of evidence-based practice. Factor analysis showed that 80.2% of the total variance of the items was explained by one variable with the Eigen values of 7.22 one part. Regarding the barriers of IEBP of participants, 243 (30.8%) of them faced mild barriers of IEBP, 195 (24.7%) of them faced moderate barriers of IEBP, and 352 (44.6%) of them faced severe barriers of IEBP.

### Implementation of evidence-based practice

The total IEBP scale score of 18 items for the participants ranged from 21 to 85. About two-thirds of the participants had a total IEBP score below 60%. From the total participants, 33.2% of nurses and midwives had a total IEBP score of 60–80% and 1.6% of them had greater than 80%. The Cronbach’s alpha for 18 items of implementation of evidence-based practice was 0.804. There was the highest mean score for the factor,” How often you read scientific articles, updated guidelines, hospital protocols, and textbooks?”, or “used evidence to change your clinical practice”, and there was also least mean score for the, “How often do you use systematic reviews report in your clinical practice?”, or “used evidence to change your clinical practice” (see Table 5). The total mean IEBP scale score of nurses and midwives was 56% and this indicated that there was a poor implementation of evidence-based practice for the total participants.

### Factors associated with the implementation of evidence-based practice

Among 790 participants, 34.8% (95% CI 31.5–38) of them implemented evidence-based practice moderately or desirably and 18.1% (95% CI 15.3–20.9) of them implemented evidence-based practice often or always. The finding of this study also revealed that 65.2% of the participants had a poor implementation of evidence-based practice. Independent variables that had a statistically significant association with implementation of evidence-based practice were the age of participants between 25 and 29 years (AOR = 5.98, with a 95% CI 1.34–26.7), poor barriers of implementation of evidence-based practice (AOR = 11.97, with a 95% CI 5.5–25.84), desirable attitude of participants (AOR = 5.02, with a 95% CI 1.2–21.5), knowledge of participants (AOR = 3.06, with a 95% CI 1.6–5.77), self-efficacy of implementation of evidence-based practice skills (AOR = 12.5, with a 95% CI 5.7–27.5), desirable nursing/ midwifery work-index (AOR = 3.9, with a 95% CI 1.4–10.87) and moderate nursing/midwifery work-index (AOR = 2.03, with a 95% CI 1.03–3.99) (See Table 6).

**Table 6:** Bivariate and multi-variables analysis of associated factors of implementation of Evidence-based practice among nurses and midwives working in Amhara Region Public Hospitals, North Ethiopia, 2019

Variables	Have IEBP		Crude OR (95%CI)	Adjusted OR (95%CI)
	Yes	No		
Age of participant				
20–24 years	38	90	1	
25–29 years	159	270	1.4 (0.9–2.14)	*5.98 (1.34–26.7)
30–34 years	51	98	1.2 (0.7–2.05)	2.8 (0.6–13.3
35–54 years	26	58	1.06 (0.6–1.9)	4.1 (0.8–20.94)
Barriers of IEBP				
Poor	159	84	***21.9 (13.72–34.97) 1	***11.97 (5.5–25.84)
Moderate	87	108	***9.3 (5.78–15.04)	***4.8 (2.2–10.6)
Desirable	28	324	1	
Attitude				
Poor	10	66	1	
Moderate	133	326	**2.7 (1.3–5.4)	1.97 (0.5–7.9)
Desirable	131	124	***6.97 (3.4–14.2)	*5.02 (1.2–**21.5)**
Self-efficacy of IEBP skill				
Poor	21	328	1	
Moderate and desirable	253	188	***21.02 (13.0–33.96)	***12.5 (5.7–27.5)
Nursing/midwifery work-index				
Poor	81	293	1	
Moderate	164	191	***3.1 (2.25–4.29)	*2.03 (1.03–3.99)
Desirable	29	32	***3.28 (1.87–5.74)	**3.9 (1.4–10.87)
Knowledge				
Knowledgeable	198	198	***4.18 (3.04–5.75)	***3.06 (1.6–5.77)
Not knowledgeable	76	318	1	

*P-value 0.05, **P-value 0.01 and ***P-value 0.001

*IEBP* Implementation of evidence-based practice

## Discussion

This study revealed that implementation of evidence-based practice was poor and it was lower as compared to other studies conducted in Ethiopia and United Kingdom [18, 24]. This could be because of the lack of knowledge, the lack of self-efficacy of implementation of evidence-based practice skills, and poor attitude towards the implementation of evidence-based practice among participants of this study. The reason for this might also be due to the lack of supporting nurses and midwives for the IEBP. The odds of implementation of evidence-based practice among nurses and midwives whose age was 25–29 years old was higher than nurses and midwives whose age was 20–24 years. This finding agrees with the study conducted in China and Norway [12, 25]. The reason for this could be young nurses and midwives whose age was 20–24 years had no experience of using evidence like guidelines, hospital protocol, research findings, and experts’ opinion. Moreover, they were newly engaged staff in clinical practice and they did not get training on different guidelines.

The odds of implementation of evidence-based practice among nurses and midwives who had desirable attitude were better than those who did have a poor attitude towards the implementation of evidence-based practice. This study finding coincides with the studies conducted in China, Oman, Nigeria, and Ethiopia [18, 25–28]. This could be because of knowledgeable respondents’ confidence in IEBP. The odds of implementation of evidence-based practice of nurses and midwives who had moderate and desirable self-efficacy of implementation of evidence-based practice skill on how to implement evidence-based practice were more likely than those who did have poor self-efficacy of implementation of evidence-based practice skill. This finding matches what was revealed in the studies conducted in Oman, Norway, and Ethiopia [12, 18, 28, 29]. This could be due to the confidence of participants to implement evidence-based practice.

The odds of implementation of evidence-based practice among nurses and midwives who had desirable attitude were better than those who did have a poor attitude towards the implementation of evidence-based practice. This is in line with the studies employed in Nigeria and China [27, 30]. This may be due to nurses’ and midwives’ positive attitude enables to implement evidence-based practice for participants of these studies. The odds of implementation of evidence-based practice of nurses and midwives who had moderate and desirable nursing/midwifery work index was better than nurses and midwives who did have poor nursing/midwifery work index. This study agrees with the result of the study conducted in Spain [31]. This could be explained by respondents who had moderate and desirable nursing/midwifery work-index could have better encouragement to implement evidence-based practice.

It was established that nurses and midwives who had poor and moderate barriers to the implementation of evidence-based practice were more likely to implement evidence-based practice than those who had desirable barriers to implementation of evidence-based practice. Factor analysis also indicated that the most important barrier of IEBP was difficulty in finding time to search for and read research articles and reports, guidelines, hospital protocols and book and having difficulty in judging the quality of research papers and reports. This finding is supported by the studies conducted in Singapore, Oman, Norway, Turkey, and Iran [10, 21, 22, 28, 32]. This is because of the lack of availability of free time to read currently available guidelines, hospital protocols, research articles, and other evidence that are important for the implementation of evidence-based practice.

### Limitations

First, the study employed in hospitals and there is more advanced human resource dynamic, quality medical service and organization structure in hospitals than the lower level of health facilities. Hence, it is difficult to generalize for all health facilities. Second, there could be socially desirable bias to the study particularly during the measurement of IEBP, self-efficacy of IEBP skills, and attitude towards IEBP.

## Conclusion

This study showed that the implementation of evidence-based practice was poor. Age of participants, barriers of implementation of evidence-based practice, the attitude of participants, self-efficacy of implementation of evidence-based practice, nursing/midwifery work index, and knowledge of participants did have a statistically significant association with implementation of evidence-based practice. Insufficient time and difficulty in judging the quality of research articles and reports are the most common barriers to the implementation of evidence-based practice. Therefore, the promotion of adopting implementation of evidence-based practice and training on the identified predictors such as knowledge, self-efficacy of IEBP skills, nursing/midwifery work index, and barriers of IEBP are mandatory.

## Data Availability

The datasets used and analyzed during the current study are available from the corresponding author on reasonable request.

## Abbreviations

IEBP: Implementation of evidence-based practice
EBP: Evidence-based practice
